# Supplementary figures and images for: Ringed seal (Pusa hispida) breeding habitat on the landfast ice in northwest Alaska during spring 1983 and 1984
Source: PLoS One. 2021 Nov 29;16(11):e0260644. doi: 10.1371/journal.pone.0260644 (PMC8629220; doi:10.1371/journal.pone.0260644)

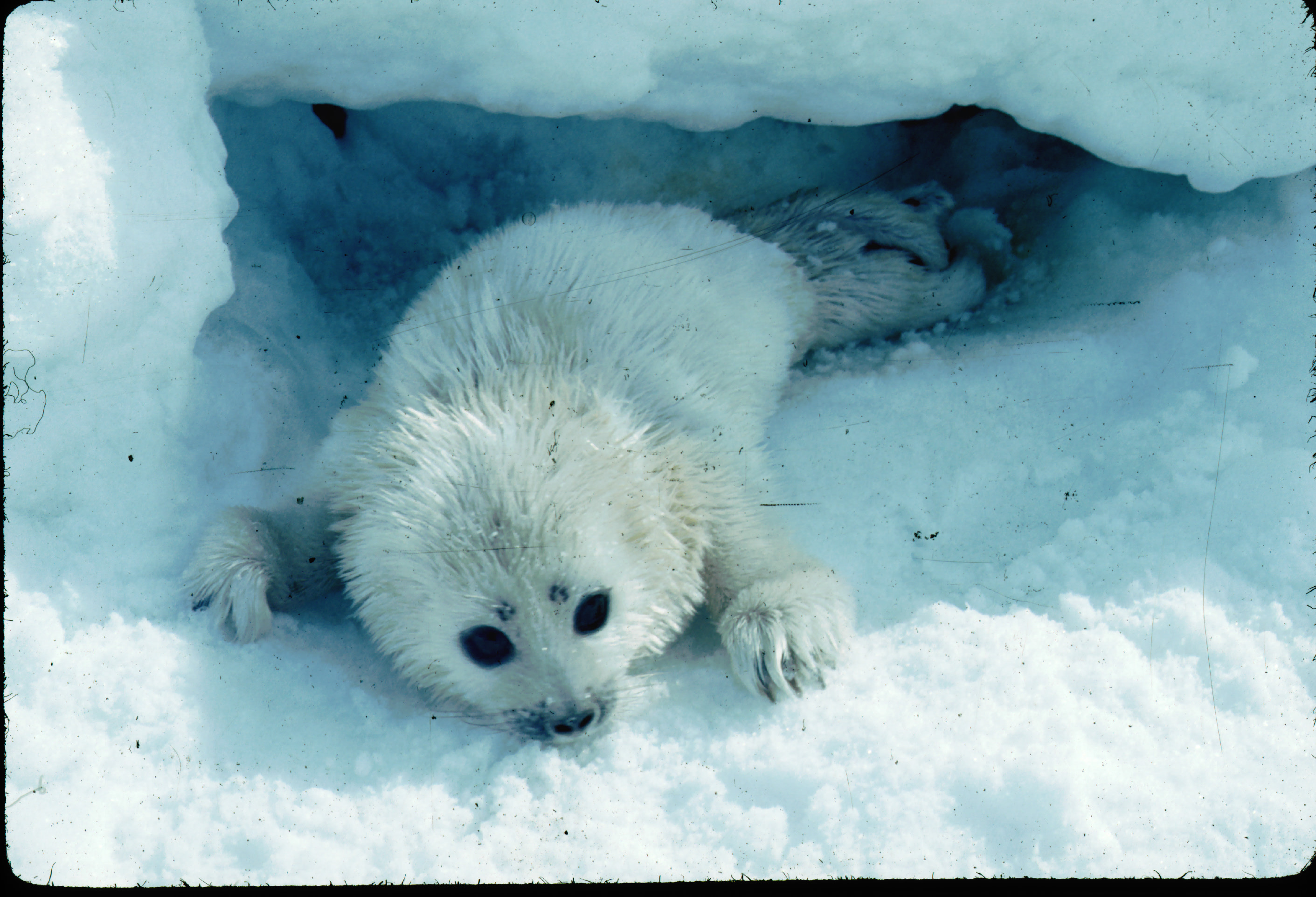

Supplement: S1 Fig — (JPG) [file pone.0260644.s001.jpg]
