# Supplementary material for: Ringed seal (Pusa hispida) breeding habitat on the landfast ice in northwest Alaska during spring 1983 and 1984
Source: PLoS One. 2021 Nov 29;16(11):e0260644. doi: 10.1371/journal.pone.0260644 (PMC8629220; doi:10.1371/journal.pone.0260644)
Supplement: Attachment — Submitted filename: Hauser2021_ReviewCrawford_20211025.docx [file pone.0260644.s003.docx]

Manuscript Review:

PLoS ONE, October 2021

PONE-D-21-28218: Ringed seal (*Pusa hispida*) breeding habitat on the landfast ice in northwest Alaska during spring 1983 and 1984

Donna Hauser, PhD.

General Remarks:

This study offers important insight into the breeding habitat of ringed seals in the Chukchi Sea. The authors have made use of an historical dataset to describe breeding habitat and describe habitat dimensions and environmental conditions, specifically, ice and snow conditions at pup lairs and other structures, during a period prior to recent large-scale climatic changes in the Pacific Arctic. They have also described the spatial and temporal variability of structure densities. These summaries provide context for current conditions.

This study will help to contribute to our understanding of breeding habitat and serve as a baseline of information prior the environmental changes resulting from climate change. These findings may also be valuable for managers, specifically those involved with determining the status of ringed seals.

It is important that this paper is published. Overall, I have no substantive concerns, but I do have some suggestions, comments, and questions that I think will improve the manuscript.

General concerns throughout the manuscript:

Throughout, change use of “haulout lair(s)” to “haul-out lair(s)”. Used this way, “haul-out” is an adjective and should be hyphenated. Other examples include haul-out data, haul-out behavior. If it were used as a noun it would be one word, “haulout”. If it were used as a verb it would be two words, “haul out”. I’ve noted a few of the instances where this needs to be changed early in the manuscript in the line-by-line comments below but I did not identify every instance. I suggest searching the entire manuscript, including figure and table captions.

Punctuation used to cite references is not consistent; sometimes they are cited using parentheses “()” and others brackets “[]”. I’m sure the copy editor would identify these instances but the authors can correct these for the next submission. Please use the appropriate punctuation for this PLOS One and be consistent through-out the paper. For example, in the paragraph starting on line 460, through 477, brackets are used 4 times and parentheses used twice. Similar to my comment above, I’ve noted a few of the instances where this needs to be changed in the line-by-line comments below but I did not identify every instance.

The order of the methods as outlined by the subsection titles and headings is a logical progression. However, some paragraphs that describe the methods should be moved to other subsections better match the progression of steps and titles of the subsections should be changed to represent the new order. See comments below.

Please note my comments regarding the proper use of the terms “digital”, “digitized”, and “digitizing” versus “plotting” and “mapping” in reference to data used in a GIS as they have very specific meanings.

Some sentences are overly verbose and could be more succinct, particularly in the Discussion. Generally, the Discussion is long and the authors should look for opportunities to make this section more succinct. However, the authors did well to compare their results to studies of ringed seals in other areas. They also framed their findings in context to observed and predicted changes in environmental conditions. Although this is appropriate and an important aspect of this manuscript, the authors should scale back their pessimism and strong language regarding predictions. While some studies have identified changes in ringed seal health associated with climate change, these changes have not been universally negative. I do think describing these predictions is appropriate. However, describing such predictions with such certainty is outside the scope of this study.

Please feel free to contact me if you have any question.

Justin Crawford

Specific suggestions and edits:

Keywords: Consider adding “survey”.

Abstract

Line 23: Change “haulout lairs” to “haul-out lairs”.

Line 24: The authors should mention the specific areas of their study before presenting results from those areas. I suggest, after “Chukchi Sea”, add “(Kotzebue Sound and Ledyard Bay)”.

Line 28: Please rewording, otherwise the reader is not sure if the grids or structures were larger in Kotzebue Sound. I suggest, “Grids in Ledyard Bay had greater overall density of seal structure than those in Kotzebue sound (8.6 structures/km^2^ and 7.1 structures/km^2^), but structures were larger in Kotzebue Sound.”

Line 35: Please rewording, otherwise it is unclear what is meant by "duration of seasonal cover". I suggest “...declines in the extent and duration of seasonal cover of landfast sea ice and snow deposition on sea ice.”

Introduction

Line 46: Change “…is…” to “…are…”.

Line 49: Please remove “and ongoing”. That Inuit people still use ringed seals as a resource is understood since the authors noted “ringed seals are”.

Line 56: This paragraph provides the biological importance of the authors’ study. Therefore, clearly describing the importance of these structures and how they are formed is critical for the foundation of this manuscript.

It widely recognized that adult ringed seals use landfast ice while most subadults do not; they use pack ice, ranging from outside of landfast ice to distances far away. I suggest you clarify this difference here, perhaps describing how these age classes use ice differently. The first sentence could describe adults, “Adult ringed seals over-winter…”. The next sentence could describe subadults, “Subadults, unconstrained by the need to use breeding habitat in stable landfast ice, use pack ice and areas near the ice edge during winter and the breeding seasons (Crawford et al. 2012)”.

CRAWFORD, J. A., K. J. FROST, L. T. QUAKENBUSH AND A. WHITING. 2012. Different habitat use strategies by subadult and adult ringed seals (Phoca hispida) in the Bering and Chukchi seas. Polar Biology 35: 241–255. doi: 10.1007/s00300-011-1067-1.

However, for the first sentence, do adults, or any ringed seals, maintain breathing holes or excavate lairs in pack ice? As I understand, adults only maintain these structures in landfast ice. As such, I suggest rewording the first sentence to clarify this, “*Adult* ringed seals over-winter in areas of landfast and dense pack ice where they maintain breathing holes *by scratching the ice* and excavate lairs in snow *that accumulates* on top of *landfast* ice [11]”.

Line 57: It’s important to clarify how the structure of ice helps accumulate snow early in the manuscript. This will help the reader understand why the authors collected the measurements and environmental data they report. Please reword, I suggest, “*Wind-drifted snow accumulates in the lee of pressure ridges and other deformations of the ice.* Sea ice with *sufficient* snow cover is necessary for construction of subnivean lairs.” Remove text after comma.

Line 59: Since the authors discuss in the Introduction and classify two types of lairs, this would be a good opportunity to quickly define them. Also, consider rewording these last two sentences to clearly describe each lair, references may need to be renumbered. As they are, they are a bit disjunct and aspects of timing for pupping lairs could be more succinct. It is also important to note that only adult females build pupping lairs. I suggest, “Lairs are classified as simple haul-out lairs, used for resting by adult male and female ringed seals, and pupping lairs, constructed by adult females to protect their pups from weather and predation [13, 21–23]. Pupping lairs are generally used during parturition in April and May, followed by 5–6 weeks of nursing, until snow melts and lair collapse later in spring [10, 20].”

Line 67: Please change “…been decreasing…” to “…decreased…”.

Line 73: Please be specific. Consider inserting “snow and ice” between “optimal” and “conditions”.

Line 79: It sounds as though you are also suggesting these conditions could affect pup survival. I suggest rewording, “*More information* is needed to elucidate the snow and sea ice conditions that influence construction of pupping lairs and pup survival.”

Line 82: Please be more direct, change “…in relation…” to “…for comparison…”.

Line 88: Please change “…haulout lairs…” to “…haul-out lairs…”.

Line 89: Please change “…in spring…” to “…during spring…”.

Line 91: The author’s second goal is to “describe the characteristics and regional composition or ringed seal structures”. Isn’t describing the characteristics of the structures the first goal? I suggest removing “…characteristics and…” from this goal and leave it as describing the regional composition. If there are other characteristics not mentioned in the first goal that warrant keeping them in the second goal, please be specific and mention them.

Material and Methods.

General comments: The order of the methods as outlined by the subsection titles and headings is a logical progression. However, some paragraphs that describe the methods should be moved to better match the progression of steps. See comments below.

Line 97: Are there “materials” than are mentioned or important enough that “materials” needs to be in the section heading? Consider renaming this section “Methods”.

Line 98: The environmental conditions described in this section are very general descriptions and do not represent the environmental covariates included in the analysis. Therefore, listing “environmental conditions” in the subsection title is misleading. Please remove “environmental conditions” and retitle this subsection “Study area”.

Line 99: The first two sentences could be more succinct and include the year each area was surveyed. Also, consider removing much of the descriptive distances; the authors provide the lat/lon and a map. Consider rewording, I suggest “Searches for ringed seal structures were conducted on landfast sea ice in two coastal areas of the eastern Chukchi Sea; southeastern Kotzebue Sound (~66˚16’N, 101 162˚32’W) in 1983 and in Ledyard Bay (~68˚53’N, 165˚50’W), east of Cape Lisburne, in 1984 (Figure 1).”

Line 103: Capitalize “Arctic”.

Line 104: Please remove, “at the latitude of the Arctic Circle”. The authors provided the lat/lons; this is sufficient description for latitude and therefore understood in relation to the Arctic Circle.

Line 106. Remove “protected and”. It’s not clear how rivers protect Kotzebue Sound. You could make the argument that, although the Sound is mostly surrounded by land that could protect the sound, there is still large expanses of open water that are not protected from wind by land. If the authors think the rivers protect the Sound, please clarify how.

Line 107: Please replace “…open northern…” to “northwest”.

Line 108: If the authors are going to introduce "characteristics of a coastal marine system" they will need to specifically mention what those are here, otherwise those characteristics are not clear. If the authors are trying to describe characteristics specifically of ice (since that is what they describe in the next sentences), they should reword this sentence to, "…Alaska coast, and the phenology and structure of the landfast ice that forms in the bay is typical of the coastal marine system of the eastern Chukchi Sea.”

Line 110: The years of the study have not been mentioned in the methods section yet. The years surveyed in each area should be added to the first sentence (or two, see my comment) that describes them.

For this sentence, 1) the authors should provide when ice formed specifically in their study area Ledyard Bay, not the Chukchi Sea coast and 2) the study is only two years. I suggest rewording, “During *both* years of our study, landfast ice *started forming* in early to mid-October in Kotzebue Sound and mid to late October in *Ledyard Bay* [39].“

Line 116: This paragraph describes the ice characteristics that allow for snow to accumulate. Therefore, it should be moved to the Introduction. I suggest succinctly combining it with the paragraph that starts on line 56.

Line 118: As written, it is unclear how sea ice dynamics influence topography of sea ice. Please edit to clarify. I suggest succinctly combining the third and fourth sentence to help clarify. I suggest, “Some pressure ridges become grounded into the ocean floor, stabilizing the landfast ice, and therefore influence the topography of the ice by generally limiting deformation of landfast ice closer to shore than on the seaward side.”

Line 120: This sentence needs restructuring to make sense. I suggest, "Wind-drifted snow accumulates in the lee of pressure ridges and other deformations of the ice." Or, if the authors want to put more emphasis on the need for structures/deformations for snow to accumulate, the authors could say, "Deformations in the ice, such as pressure ridges, are necessary for wind-drifted snow to accumulate in the lee of these deformations, particularly for snow to accumulate to depths sufficient to support ringed seal liars."

Line 123: This subsection should be titled “Detecting ringed seal structures”. This is a lengthy subsection and it makes more sense to include the classification process with the measurements in the next subsection.

Line 124: The sentence providing the dates of searches should be moved to the first paragraph in the Methods. I suggest making this the second sentence of the first paragraph.

Line 127: Please clarify what is meant by “[following procedures of 8]”. Is this in a reference?

Line 128: Please replace “trotting in front” with “ahead”.

Also, the authors mentioned the surveys were along “pre-established survey lines”. Please provide more information about the survey lines. Were they random, gridded, at specified distances from each other, parallel, a specified length? Knowing the design of the surveys will help interpret the results of the surveys. See comments below regarding survey design on line 199.

Line 133: The reader should know that the description of measurements and classifications for structures will be defined later in the methods, else it is unclear if they were omitted. After “classified”, please insert “(see below for measurements and classification definitions)”.

Line 135: This paragraph should be included in the next subsection; it makes more sense to describe the classification with the measurements. Please move to the second paragraph of the next subsection, renamed “Measurements and classification of seal structures”.

Line 135: Please replace, “There were four types of structures:…” with “We classified four types of structures:…” or “Four types of structures were classified:…”.

Line 150: Please reword this subsection title to follow the order in which these steps were taken while at the structure. Reword to “Measurements and classification of seal structures”.. This is clearer.

Line 156: Please remove extra verbiage, “in contrast to”. I suggest, “…pressure ridges that facilitate snow accumulation; lower values indicate flatter, …”

Line 158: The authors mention a “range of deformation”. In this sentence, should this be “ice relief” if the measurement is a height/distance and not a percentage? Ice deformation is described in the following sentence.

Line 159: Please provide detail for how the percentage of ice deformation is determined and, if it will help explain the process, an example. Is it similar to percent cover estimates where the percentage represents the aerial proportion of ice that is deformed (not flat)? Please clarify.

Line 161 and 178: These two paragraphs describe the survey process for location seal structures and should come before the measurements and classifications. Please move these two paragraphs into the previous section, renamed “Detecting ringed seal structures”. These two paragraphs should be the second and third paragraphs in that subsection. Authors may need to edit these paragraphs to remove redundant information given in the first paragraph of this subsection.

Line 181: The wording is awkward and unclear. Please clarify, I suggest, “Despite the perception that wind affects a dog's ability to locate structures, the dogs were equally...”.

Line 185: The segment, “with the wind on each side,” is not clear. Do the authors mean “with the wind originating on each side”, such that they moved along the line in both directions? If so, I suggest, “Each grid line was searched at least twice, *traversed in both directions to minimize the effect of wind direction*, and most lines were searched repeatedly until no additional structures were found.”. If not, please clarify as is this is not clear.

Line 190: GISs are technology; therefore “technological” is redundant. Please remove “technological”.

Line 191: Reword to clarify. I suggest, “…accurate mapping *and assessment* of the spatial distribution…”. Mapping “of” the spatial distribution is redundant; by mapping these structures that act is inherently creating a spatial distribution. Mapping these data allows you to assess the spatial distribution. Also, please remove “digital”, see below.

Also, it appears the authors use the terms “digital”, “digitized”, and “digitizing” repeatedly. The process of “digitizing” spatial data has a very specific meaning. Digitizing was used in the early days of GIS when programmers would use a board and mouse-like tool to convert data directly from maps (outlining) to a GIS. From the methods provided and my experience with GIS, it does not sound as though that was the process the authors used. It sounds as though the authors created points from lat/lons; i.e., created a shapefile from a spreadsheet or manually typing lat/lons into a GIS. This process is referred to as “plotting” or “mapping” and the authors should simplify their descriptions throughout the manuscript by removing references to the terms “digital”, “digitized”, and “digitizing” and use "plotted" or "mapped".

However, if I have misunderstood and structure locations were digitized using the old method of scrolling a mouse on top of a georeferenced map, these methods should be described in more detail and clarified.

Line 193: Remove “digitize and”. Stating “to map” is sufficient.

Line 194: Please reword and remove “Digitizing”. Also, the phrase "Shapefile points" for known “points” is somewhat redundant (yes, shapefile can be points, lines and polygons but this phrasing is still awkward). I suggest rewording, “Plotting each grid involved first creating a shapefile for the known anchor points.".

Line 196: ‘Create Fishnet’ is a relatively new tool in GIS. Please include the toolbox or references (e.g., Data Management toolbox).

Line 197: Remove “digitized”.

Line 199: In reference to the “440 yard staking”, does this correspond the edge of each grid (which is implied by the text), or did the staking represent the midpoint of each cell such that the actual transect was along that stake (midpoint of the cell/grid) and the cell itself represented the effective survey strip width? This is unclear and should be clarified. If the later is true, this sentence should be reworded.

Line 202: Remove “digitized”.

Line 203: Remove “digitized” and replace with “plotted”.

Line 206: Similar to above, “point shapefiles” is somewhat redundant and awkward. I suggest, “We also created a shapefile of the locations of all seal structures.”

Also on line 206, locations are inherently geographic and therefore saying “geographic locations” is redundant. Remove “geographic”. This is the first point in the manuscript the authors have used the term “vertices”. Since this study is not saturated with GIS methods, I suggest describing these in terms of the grid; I assume vertices refers to the intersection of grid lines. I suggest succinctly rewording, “*To estimate structure locations*, we first created points for the *intersection of grid lines*, which were manually labeled…”

Line 209: Geographic is implied with coordinates. Remove “Geographic” and start the sentence with “Coordinates”.

Line 212: Please be specific. Insert “survey” to read, “…corner of a *survey* block to the location…”

Line 211: Please provide the toolbox for the “Bearing Distance to Line’ tool.

Line 214: In reference to the “environmental measurements and attributes”, please clarify that these were collected in the field and not by another process. I suggest rewording, “Lastly, associated environmental measurements and attributes sampled in the field were appended to plotted seal structures.”

Line 216: Remove “other”, it is implied.

Results

Line 248: Replace “..was…” with “…were…”

Line 252: Replace “..greater..” with “..higher..” “Greater” implies one was better or worse.

Line 257: Insert an “a” before “generator”.

Line 260: Replace “..greatest..” with “..highest..”

Line 264: The authors noted that the most common structure in both regions were haulout lairs (0.41). Was 0.41 the proportion for each region or was that the overall study area (combining both regions)? In Table 2, haul-out lairs accounted for 0.40 of structures in Kotzebue Sound. Please clarify. Since the sentence, the proportion of pup lairs, reports the proportions from each study area, I suggest being consistent and report both for haulout lairs, “The most common structures in both regions were haul-out lairs (> 0.40, Table 2).” Alternatively, the authors can reword the sentence to report the proportion for the overall study, “The most common structures in the overall study area (combining the two regions) were haul-out lairs (0.41, Table 2).

Line 265: Please use “haul-out” lairs. Replace “..greater..” with “..higher..”

Line 266: The authors are comparing Kotzebue S. and Ledyard Bay. They should explicitly state this. I suggest rewording, "Within Kotzebue Sound, the proportion of pup lairs..."

Line 268: For this comparison, please either reference Table 2 or provide the proportion of simple breathing holes found in each study area.

Line 276: Consider specifically referencing Figure 3a after the p-value.

Lines 276–280: The authors report the diameter of haul-out lairs twice. These sentences should be more succinct. I suggest rewording, combine these sentences, and try to reduce redundancy, “Overall, simple breathing holes were smallest (mean diameter = 29.8 cm), followed by haul-out lairs (mean diameter = 38.3 cm), and pup lairs had the largest access holes (mean diameter = 48.2 cm)”.

Line 280: Replace “Area” with “Structure area”. This variable name is consistent with the label on the Y axis of Figure 3b and is more specific. Please use “haul-out lair”.

Line 282: Consider specifically referencing Figure 3b after the p-value. Please use “haul-out lair”.

Line 283: Replace “..greater..” with “..longer..”

Line 284: Replace “..greatest..” with “..largest..” Also, please be more specific; it’s unclear what the authors are reporting by “greatest area”. I suggest rewording, “The largest area of a single structure (15.1 m^2^) was measured…”

Line 288: Consider specifically referencing Figure 3c after the p-value. Also, please provide a statistic of variability that supports height being the “least variable measurement”.

Line 289: Replace “In” with “For”.

Line 290: Regarding the authors statement that structures were larger in Kotzebue Sound than Ledyard Bay, please provide a summarized P-value or other statistics to support this. I recognize this statement covers multiple comparisons, however, the authors can present the p-value for the least significant comparison and note that all comparisons were less than it; e.g., (p < 0.031 for all comparisons).

Line 293: Consider specifically referencing Figure 4a after the p-value. Also, is there a reason the authors did not report the overall p-values for snow and ice? In the next sentence they mention "in general", which implies an overall comparison.

Line 294: Replace “..greatest..” with “..deepest..” Also, the authors report snow depths were deepest at pup lairs in Kotzebue Sound but what about Ledyard Bay? Describing this difference in the following way puts more emphasis on the differences, which I think readers are more interested in. Please reword to something similar to "In general, snow depth was *deepest* at pup lairs, however, snow at pup lairs was only significantly deeper than *haul-out* lairs in Ledyard Bay (p = ??; Kotzebue Sound, p = 0.085).

Line 295: Replace “..greater..” with “..deeper..”

Line 297: Replace “..greatest..” with “..deepest..”

Line 300: What do the authors mean by altered? Was it abandoned as a pup lair and only used as a breathing hole during the study? If this was still used as a pup lair, can the authors clarify, perhaps mentioned why, for example ", although the roof on this lair was open.." Please clarify, otherwise it sounds as though the authors arbitrarily changed the status of this structure.

Line 301: Consider changing “unaltered” to “intact”. Also, consider removing “with evidence of a pup” from this sentence. This text is not necessary since “evidence of a pup” was a requirement mentioned in the Methods. Also, it makes me wonder why those same criteria were not mentioned in the next sentence for pup lairs in Kotzebue Sound, or elsewhere.

Line 301/2: Consider combining these two sentences to be succinct and easier to read. I suggest, “Minimum snow depth for intact pup lairs was 47 cm in Ledyard Bay and 58 cm in Kotzebue Sound."

Line 304: This sentence is overly wordy. Consider removing, “it was” and “it”, leaving “…among structure types in Kotzebue Sound (F = 1.92, df = 2, p = 0.149), but in Ledyard Bay (F = 5.98, df =2, p = 0.0029) it differed between breathing holes …”.

Line 305: Consider specifically referencing Figure 4b after the p-value.

Line 305: The authors report “Mean deformation was similar in both regions.” I suspect this was pooling across all structure types, but this is unclear? Or, was this deformation for a specific structure type? Please clarify. If the authors are reporting a pooled mean, they should clarify and reword to something similar to, “Pooling among all structure types within each region, mean deformation was similar in both regions (26–29%, P-value).” Also, please provide a p-value or appropriate statistic for this comparison.

Line 307: Replace “in either” with “within each”.

Line 308: Consider specifically referencing Figure 4c after the p-value for the sentence ending on this line.

Line 308: Replace “..greater..” with “..higher..” Also, please provide a test statistic supporting this difference.

Discussion

The authors should look for opportunities to make this section more succinct.

Line 313: The description of the process of structure construction, given in this first sentence, from breathing hole, to snow accumulation, to excavation, should be included in better detail in the Introduction (see comments). For detail, I suggest including that snow must drift over the holes. Consider rewording to, “All lairs begin as breathing holes which, once drifted snow has accumulated over the holes, are then excavated into simple single-chambered lairs and are later enlarged to include multiple chambers when a pup is present.”.

Line 314: The sentence reporting the “irregularities” should be moved to the end of this paragraph or put in a new paragraph. Discussing these “irregularities” this early in the Discussion is confusing.

Line 325: When reporting the area sizes, replace the hyphens with equal signs.

Line 328/9: Please reword this sentence. I suggest, “Ringed seals in both the eastern Beaufort Sea and Svalbard *regions were longer* than those in the eastern Chukchi Sea”. Both areas are regions and the past tense should be used; these measurements were taken roughly 40 years ago. Also, the authors are referencing seal length; therefore “longer” is more appropriate than “larger”.

Line 331: Please replace “is” with “was”. The past tense should be used; these measurements were taken roughly 40 years ago.

Line 333: At the end of this sentence, consider replacing “diameter” with “girth”. This change makes the connection to the following sentence tighter.

Line 333: In reference to the sentence that starts “Although”, I presume “smaller” in this sentence refers to girth since the authors have already discussed length. If so, please be specific and clarify. Also, past tense. I suggest, "Although Alaska ringed seals *had* smaller *girths* than seals in other areas [43]... "

Please clarify the rest of this sentence. It is not clear which “diameter” you are referring to; diameter of the hole or diameter of the seal (or possibly the height of the lair)? I assumed the authors mean the seal diameter, since it was referenced in the previous sentence, but girth measurements are diameter measurements, which confuses the rest of the sentence. Please clarify. I think the authors are trying to suggest changes in seal girth do not affect the lair height as much as seal length affects lair width/length. I suggest changing the end of this sentence to clarify, "...[43], a substantial difference in seal girth *would not substantially affect lair height*."

Line 335: Word choice make this sentence awkward. Please change the beginning of this sentence to read, “Difference in methods among studies…”.

Line 336: Here, the authors discuss differences in how lairs were measured among studies. This discussion would be more informative if the authors also mentioned how they think those differences may affect differences in measurements or interpretation. Are they suggesting the methods in this study may result in more variability in measurements than those in other studies? Please clarify.

Line 345: The beginning of this sentence is awkward. Please reword, I suggest, “*Parameters used to classify lairs* likely affects whether differences are observed among lair types”.

Line 346: Is there an extra space between sentences?

Line 347: This sentence starts by explaining the criteria for classifying a pup lair. However, the authors also mentioned in the Methods that they also noted evidence of pup use. Please add to this sentence. I suggest, "...assumed to be used by females with pups*, and often confirmed by observing evidence of pup use,* while in *other* studies..."

Line 350: Please be specific about “what they signify”. I suggest replacing “what they signify” with “the interpretation of the significance of those distances”. Also, remove “Reports of” at the beginning of the sentence so it starts, “Distances between..” The reader is not concerned about the “reports”, we are interested in the distances.

Line 351: I suggest you remove the sentence starting “In this study we noted..”. The reader already knows this from the Methods and it does not help interpretation in this paragraph.

Line 353: Please reduce redundancies. Please remove, “For both of our study areas combined, “ and start the sentence “The overall mean…”

Line 355: This information is not in the Results, but should be. Please move the sentence that starts, “Pup lairs were located…” to the results section, in the paragraph covering distances. Please use “haul-out lairs.”

Line 363: "Tagged" seals has multiple interpretations. Since your study is not a biologging study and this is the first reference to "tagged" individuals, the authors should use terminology similar to what the authors of 35 used and mention where these seals were monitored. Please replace “tagged seals” with "radio-tagged seals in the Beaufort Sea".

Line 364: Remove “also”.

Line 367: Please remove the sentence that starts, “One female…” The authors already mentioned seals use multiple lairs.

Line 367: Please replace “tagged” with "radio-tagged".

Line 369: Wording is awkward. I suggest rewording, “We did not *instrument* seals in this study *and therefore cannot assess* the number of structures...”. Also, consider replacing “tag” with “instrumented”.

Line 371: Please insert “individual” before “seals”.

Line 373: Please remove the first instance of “and”. This should read, “… seal, prey availability, as well as…”

Line 376: Please be succinct. I suggest rewording, “*Most* studies of ringed seal pupping habitat *report a* mean snow depth *at* pup lairs of 65–80 cm*,* 60 cm at haul-out lairs, and 35–45 cam at breathing holes.”

Line 381: Please reword, replacing “in” and “greater”. I suggest, “In this study, lairs were located *on* ice with *more* deformation…”

Line 382: Replace “..greater..” with “..more..”

Line 387: To be consistent, please replace “..structures..” with “..structure types..” (used in next sentence). Also, remove “geographic”; this is redundant with “regions”.

Line 388: Please be succinct. It’s not that there was variation, the proportions were different. Also, this is the first reference to “smaller grids.” If the authors think this distinction is important, they should clarify what defines smaller grids and why they think this is important. I suggest rewording, “However, the proportions of structure types were *different among the individual* grids within regions, …”

Line 390: Replace “..greater..” with “..more..”

Line 391: Please be succinct. Please replace “had a large effect on” with “substantially affected”.

Line 393: I suggest rewording, “The composition of ringed seal structures varies considerable across its range (Table 5)". The Discussion is long. The authors should try to be as succinct as possible to shorten the Discussion.

Line 395: Past tense. Replace “is” with “was”.

Line 395: In reference to the “size” of study areas, discussing the size of the study area may not be intuitive and is not clear. The authors should clarify this. I suggest adding a sentence to similar to, "Large study areas tend to include large areas of flat, undeformed ice that do not accumulate snow and therefore are generally not suitable for breeding habitat (i.e., pup lairs)."

Line 396–398: Especially because this paragraph discusses “geographic variation” across the range of the ringed seals, the authors should rephrase this statement to put the emphasis on the location, not the researchers. Please reword this sentence similarly to, "For example, in the White Sea, 9% of structures across an entire study area were pup lairs. However, the entire study area contained large areas of flat ice and habitat not suitable for lairs. In areas with adequate snow cover and ice deformation (prime breeding habitat), 33% of all structures were pup lairs [47]." Be consistent with citing, “[]” or “()”.

Line 402: Please insert “and type” after “number”.

Line 410: Insert a comma after “In this study”. These two sentence (410 and 411) are somewhat awkward and could be more succinct. I think I see the point the authors are trying to present but I think it can be clearer. I suggest, "In this study, dogs searched survey grids along lines spaced such that the dogs could thoroughly survey the entire grid". The distance of the spacing (~400 m) was mentioned in the Methods.

Line 418: Please reword to clarify this thought. I suggest, "... , it is not possible to distinguish differences in the availability of suitable breeding habitat between regions from natural inter-annual variability".

Line 420: Please add commas to make this sentence easier to read. I suggest, “Density differences can also be due to, or augmented by, factors such as prey availability.”

Line 422: Please insert a comma after “density”.

Line 424-5: Please provide the year for each description. For example, “In 1985, seal densities were higher in Ledyard Bay, in 1986 they were lower, and in 1987 they were similar to 1985.

Line 425: Similar to previous comments, I think the emphasis should be on the location and not the researchers for this study. I suggest rewording, "In the White Sea, Lukin and Potelov (47) observed a 30% reduction in structure density between 1972 and 1973." Also, can the authors clarify which structures were reduced in density? Was the reduction only for lairs or was it for all structures?

Line 426: Similar to the previous sentence, where were these observations? I suggest rewording to put the emphasis on the location, "In the western Beaufort Sea, Smith and Stirling (8) found one pup lair per 6 minutes of searching in 1973 *and* to 1 per 64 minutes the following year."

Line 428-9: Please remove “It is clears from both” and cleanly start this sentence, “On-ice studies…” Please insert, “both suggest” after “seals”. Also, replace “geographic” with “spatial”; spatial is more appropriate here. To clarify, I suggest rewording, “*On-ice* studies of seal lairs and aerial surveys of hauled out seals *both suggest* that ringed seal habitat is dynamic and highly variable on both temporal and *spatial* scales.

Line 431: Remove “geographic”. “Variations in environmental conditions” are not necessarily always geographic.

Line 433: Between “since” and “1983”, I suggest inserting “the time of our surveys in”. Also, insert “sea” before “ice”.

Line 452: Replace “geographic” with “spatial”; spatial is more appropriate here.

Line 460–548: Topics from this and the next 5 paragraphs do not necessarily associate with the subsection, “climate change”. I suggest the authors either move these paragraphs to the subsection on characteristics of structures or give them their own subheading.

Line 460: I suggest swapping the order of “snow accumulation” and “ice deformation”, since ice deformation occurs prior and is necessary for snow to accumulation.

Line 462: It sentence represents an important component of management of ringed seals in the context of climate change. As such, after “pup survival”, I suggest inserting, “, despite its perceived importance for management”.

Line 470: This sentence has issues with readability. I recommend the authors either remove "reported as" and end the sentence, “in the early 1970s was 25 cm”, or reword the sentence to be more readable. I suggest, "The minimum snow depth for pup lairs was 25 cm in the eastern Beaufort Sea in the early 1970s (8).

Line 471: insert “et al.” after “Stirling”.

Line 480: I assume the authors are referring to physical snow characteristics. To clarify, please provide and cite these characteristics. For example, cohesion.

Line 483: Remove “reported”. Mean snow depth may suffer from the same issues as minimum snow depth. If all the lairs are measured at the same time, during late spring, presumably all the snow will have melted and depth condensed similarly, affecting the mean depth. Perhaps measuring should take place before melt and condensation takes place, else it should be noted.

Line 485: By “greater integrity”, do the authors mean greater “structural integrity”. This should be clarified, as well as other factors that affect structural integrity. While I suspect snow depth does influence structural integrity, it is not the only characteristic of snow affecting structural integrity and may not be the most important one (although I recognize depth’s importance for lair construction for seals, these are separate arguments). The authors should provide a reference for this assertion. If the authors do not have a reference, this sentence can be removed without loss to the paragraph. The next sentence associates snow depth with predation success, which is referenced, and important on its own.

Line 486: Polar bears also prey on bearded seals. Please be specific and mention ringed seals. Also, this sentence has issues with readability and could be more clear. I suggest, “....polar bear predation of ringed seals was less successful and fewer attempts on known lairs occurred in area of deeper snow []."

Line 493: This argument could be clearer and ordered more succinctly. Consider rewording to emphasize the pup's ability to sufficiently insulate itself. It is not as much the actual age of the pup as it is the pup's ability to sufficiently insulate itself, which comes with age. I suggest, “Overall, the timing of snow melt, the integrity of the lair, and the pup’s ability to sufficiently insulate itself are important factors for understanding minimal snow depths sufficient for pup survival.”

Line 504: Try to keep this argument in the context of the season, i.e., earlier in the season. I suggest rewording, "... other grids that were surveyed earlier in the spring." Also, I suggest numerating the study grids by inserting “five” before “Ledyard Bay”.

Line 507: I suggest moving this paragraph to the second-to-last paragraph of the Discussion. This is a well organized summary statement.

Line 515: It is incredibly important that the authors mentioned that not all ringed seal use subnivean lairs. Consider mentioning that these other conspecifics and their breeding habitat are at the southern range of ringed seals' distribution. This is an important distinction in the context of predicted effects of climate change for more Arctic subspecies.

Line 517: Capitalize “Arctic”.

Line 529: The authors have set up a productive discussion with this paragraph. By mentioning two areas where ringed seal health appears to be different, they have highlighted that seals may respond differently to environmental changes. However, I think the authors should explicitly mention this before discussing the two studies.

I suggest inserting the following before the sentence discussing ringed seals in Alaska, “Across their range, ringed seals, as well as their primary predator [a, b below], may have different responses to environmental changes.” I also suggest referencing these two studies because they help reinforce the idea that changes are having negative effects regionally and not necessarily across the Arctic.

a - Stirling, I. (2002). Polar bears and seals in the eastern Beaufort Sea and Amundsen Gulf: A synthesis of population trends and ecological relationships over three decades. Arctic, 55, 59– 76. https://doi.org/10.14430/arctic735

b - RODE, K. D., E. V. REGEHR, J. F. BROMAGHIN, R. R. WILSON, M. ST MARTIN, J. A. CRAWFORD AND L. T. QUAKENBUSH. 2021. Seal body condition and atmospheric circulation patterns influence polar bear body condition, recruitment, and feeding ecology in the Chukchi Sea. Global Change Biology 27: 2684–2701. doi: 10.1111/gcb.15572.

Line 533-5: I don't think this assessment of pups harvested is necessary for this paper and is somewhat outside the scope of your paper. Consider deleting these two sentences. The point Harwood and others have made is that by itself, the proportion of pups harvested may not be the best index of reproductive success but, in conjunction with other measures, including ovulation or pregnancy rates it can inform our understanding of a populations “health”. Although there are caveats with the proportion of pups harvested and relatively small changes in this index are likely a product of these caveats and should not, by themselves, be considered population level changes, this index does gauge whether pups have failed to be weaned, and are therefore not available to be harvested, or were successfully weaned, and therefore has merit.

Line 552: The discussion of the UME is outside of the context of this study (breeding habitat), especially since the causes of both UMEs in Alaska have not been determined. The points raised at the beginning of this paragraph, up to line 552, have merit and could be incorporated into the previous paragraph or stand alone. However, I suggest removing discussion of the UME, from line 552 to the end of this paragraph.

Line 564: The suggestion that breeding habitat may be unsuitable for successful pupping is too strong a statement. The authors report on data collected nearly 40 years ago. There are more recent studies that suggest ringed seals in the same region as those studied by the authors have yet to be negatively affected by climate changes. Further, if breeding habitat in the Bering and Chukchi seas is predicted to resemble habitats more similar to those at the southern range of ringed seals where they pup on ice and beaches (not in lairs), which may be less productive, ringed seals will like continue to reproduce but at a lower rate, making this statement too strong. The Pacific Arctic may not have reached a threshold, beyond which environmental changes would negatively affect ringed seal. However, even if changes reduce pupping success it is unlikely that the population would experience population-wide reproductive failure. Therefore, this statement is too strong.

Also, consider rewording to remove “like” and be specific. I suggest editing this sentence to something similar to, “Recent studies suggest that Arctic regions, such as the Bering and Chukchi seas, are undergoing rapid ecological transition [70], and therefore breeding habitat in this region may be altered.”

Figures and Tables:

Figure 1, line 775: For the main, study-wide map, 1) label Ledyard Bay, and 2) the map should pan out/back more to include more of Ledyard Bay; for one of two study areas it is barely included on the map.

Survey grids: 1) the scale and north arrow are too small and not readable, and 2) the small study area insets are not necessary for the survey grids since they are also identifiable on the main study-wide map. I suggest removing these small insets, however, if they are kept, they should be larger (they are too small to be readable) and the grid being identified should be outlined with a more contrasting color to be obvious, maybe red. Removing the insets would provide more space to increase the size of the scale to make it readable.

Figure caption: Consider rewording to be more succinct and add the years areas were surveyed. I suggest “Study areas in Kotzebue Sound (1983) and Ledyard Bay (1984)*, including maps of survey grids and all ringed seal structures located by dogs.*” The readers already know you used a GIS, therefore it is clear these were digitally mapped; no need to qualify this statement with “digital”.

Figure 3, line 788, figure caption: Consider rewording to be more succinct and split caption into two sentences. All plots visualize comparisons and show “differences” (if they exist). Please note what is being summarized. I suggest, “Boxplots *summarizing dimensions* of seal structures (B = breathing hole, H = simple haul-out lair, P = pup lair) in Kotzebue Sound (KS) and Ledyard Bay (LB). *Dimensions include* a) diameter of breathing holes, b) area of structures, and c) height of structures.” Please use “haul-out lair”.

Figures 3 and 4: Green is often not visible for colorblind people. Please change these colors to a contrasting pair of colors that are visible for the colorblind (for both figures 3 and 4). Colorblind-safe colors can be found at <https://colorbrewer2.org/>

For both figure captions, the parts of boxplots are often used to depict different statistics. While it is most common for the mid-line to represent a median it can also represent the mean. Please identify what the mid-line, box, whiskers, and dots represent in both figure captions.

The authors reference and make comparisons for overall measurements in the text. Please add lines to figures 3 and 4 that represent these overall measurements? For each subfigure, it could be a horizontal or line, of a different and contrasting color or dashed, that spans each B, H, P structure.

Figures 3 and 4 and Tables 3 and 4: The authors have done well to make these captions consistent. However, they were not consistent in how they defined “H” in each; in Figure 3 the authors identify “H” as a “simple lair” while in Figure 4, Table 3, and Table 4, “H” is identified as a “simple haul-out lair”. Please be consistent and use the same definition in all captions and please use “haul-out lair”.

Figure 4: For all three of these plots, there are data being shown beyond the y-axis. Please fix this. It's probably easiest to expand the limits of the y-axes to include the maximum data-points.

Line 792, figure caption: Similar to Figure 3, consider rewording to be more succinct and split caption into two sentences. I suggest, “Boxplots *summarizing environmental conditions at* seal structures (B = breathing hole, H = simple haul-out lair, P = pup lair) in Kotzebue Sound (KS) and Ledyard Bay (LB). *Environmental conditions include* a) snow depth at the structure, b) extent of ice deformation, and c) ice relief height.” Please use “haul-out lair”.

Table 1: In the table caption, please identify what the lat/lons represent; e.g., the middle of the grid, the closest corner to land, or something else.

Table 2: Fix the formatting on the right side of this table, vertical lines and protruding bars should be removed.

Field headings: Please use “Prop. haul-out lairs”.

Within Table 2, row 83-2, column “Density of structures”: Is there a reason this doesn't equal 9.71 (57/5.87)? If so, please explain/footnote, else correct.

Within Table 2, row Total (Kotzebue Sound), column “Density of structures”: Similar to above comment, should this be 242/33.42 = 7.28, not 7.07? If there is a reason this is different please explain/footnote, else correct.

Table 3: In the caption, the authors described the data being presented in each cell. The authors do not need to include "n=" in each cell, please remove “n=”. Also, it appears the authors italicized most sample sizes but not all. Italicizing the sample sizes would help identify them. Please be consistent with italicizing through the table.

In the caption, please use “haul-out lair”.

Within Table 3, row Height (cm), column “Regions combined, H”: Please force the SD (8.4) to the line under the mean so each cell has identical format throughout the table.

Within Table 3, row Area of lair, first four columns with data”: Please force the SD (#.#) to the line under the mean so each cell has identical format throughout the table.

Table 4: For the range of minimum-maximum values in the Ice relief heigh row, please confirm these data. It is odd, but certainly not impossible, that 5/6 ranges are identical, “31–183”.

In the caption, please use “haul-out lair” or “simple haul-out lair”.

Table 5, caption: The caption should be more descriptive than only describing "parts of their range", especially since some of the data presented are from other studies. Consider changing to “Comparisons of mean (minimum-maximum) measurements of ringed seal lair characteristics and environmental conditions from surveys conducted on the landfast ice during the pupping season using trained dogs. These summaries include data collected by this study as well as data from studies in the Beaufort Sea, Canadian Arctic, Svalbard, Norway, and Russia.

Table 5, left most column: Please include units where appropriate: Breathing hole diameter (??), Lair height (??), Lair length (??), Lair width (??), Lair area (??), and Distance to nearest structure (??).

Table 5, left most column: Please be consistent with these labels where appropriate; it makes sense not to add “lair” for the four variables specific to lairs (height, length, width, and area). Please be consistent with naming. In the first group the authors use “Haul-out lairs”. “Lairs” should be added to “Haul-out” and “Pup” in the second group, “Breathing hole diameter” and the last group, “Distance to nearest structure” as these groups are not specific to “lairs”. Please rename the second group, “Breathing hole diameter” to “Hole diameter”. It is somewhat confusing to have a structure name match the group name and haul-out and pup lairs do not use the hole only for breathing. “Hole diameter is sufficient”. Please use “haul-out lairs”.

Table 5, footnote “a”: Please reword footnote “a” to be more succinct; 9% is already in the table and is not necessary. I suggest “Researchers included flat ice and areas with little snow cover in their survey of available habitat. When areas of...”

Table 5, last line of footnotes: Should “Birth lairs only” be labeled with a “c”?
